# Using phage Lytic Enzymes to Control Pathogenic Bacteria

**DOI:** 10.1186/1472-6831-6-S1-S16

**Published:** 2006-06-15

**Authors:** Vincent A Fischetti

**Affiliations:** 1 Rockefeller University, New York, NY 10021, USA

## Abstract

Our laboratory has developed phage lytic enzymes to prevent infection by specifically destroying disease bacteria on mucous membranes and in blood. Enzymes specific for *S. pneumoniae *and *S. pyogenes *have been developed to be used nasally and orally to control these organisms in environments such as hospitals and nursing homes to prevent or markedly reduce serious infections by these pathogens. In addition, a *B. anthracis*-specific enzyme was developed to kill the vegetative forms of these bacteria in the blood of infected individuals. In animal studies, >80% of mice colonized mucosally or infected intravenously with pathogenic bacteria were decolonized or survived after a single enzyme treatment delivered to the same site of colonization or infection.

## Introduction

Bacteriophages infect their host bacteria to produce more virus particles. At the end of the reproductive cycle (which may last up to an hour) they are faced with a problem, how to release the progeny phage trapped within the bacterium [1]. They solve this problem by producing an enzyme called lysin that degrades the cell wall of the infected bacteria to release the progeny phage. The lytic system consists of a holin [1] and at least one peptidoglycan hydrolase, or lysin, capable of degrading the bacterial cell wall. Lysins can be endo-beta-N-acetyl-glucosaminidases or N-acetylmuramidases (lysozymes), which act on the sugar moiety, endopeptidases, which act on the peptide cross bridge, or more commonly, an N-acetylmuramoyl-L-alanine amidase (or amidase), which hydrolyzes the amide bond connecting the sugar and peptide moieties. Typically, the holin is expressed in the late stages of phage infection, forming a pore in the cell membrane, allowing the preformed lysin(s) to gain access to the cell wall peptidoglycan, resulting in release of progeny phage. Significantly, exogenously added lysin can lyse the cell wall of healthy, uninfected cells, producing a phenomenon known as lysis from without. However, because of the lack of an outer membrane, this event is observed only in gram-positive bacteria.

While lysins have been known for many years [2-5], our laboratory is the first to use these enzymes therapeutically and prophylactically *in vivo *in their purified form to kill colonizing pathogenic bacteria on mucous membrane surfaces and in blood. We have been successful in using such enzymes to kill *S. pyogenes *[6,7] and more recently, *B. anthracis *[8]. All of these enzymes are highly evolved molecules designed for a specific purpose, to quickly destroy the bacterial cell wall, and as such, we find that nanogram to sub-microgram quantities of enzyme per milliliter is sufficient to sterilize a 10^7 ^bacterial suspension in seconds. To date, other than chemical agents, there is no biological compound known that can kill bacteria this quickly. Such phage lytic enzymes are the culmination of millions of years of development by the bacteriophage and its association with bacteria. Since nearly all bacteria are or can be infected by bacteriophage, such enzymes may be developed for nearly all disease-causing bacteria. It appears, however, that these enzymes work best against gram-positive bacteria. In these organisms, the enzyme is able to rapidly access the bonds in the peptidoglycan when added extrinsically. In the gram-negative bacteria, the outer membrane is a barrier for these enzymes.

### Enzyme Structure

A feature of those phage lytic enzymes that have been characterized so far is their domain structure [9,10]. Generally, the N-terminal domain contains the catalytic activity of the enzyme, which will cleave one of the four major bonds in the peptidoglycan of the bacterial cell wall as described above [1]. Of the phage lytic enzymes that have been reported thus far, the great majority are amidases. The C-terminal domain of phage lytic enzymes has specificity for a cell wall substrate [11-14]. Thus, unless the binding domain binds to its wall substrate, the catalytic domain will not cleave, offering specificity to most enzymes studied. The reason for this specificity was not apparent at first, since it seemed counterintuitive that the phage would specifically design an enzyme that was lethal for its host organism. However, as we learned more about how these enzymes function, the possible reason for this specificity became apparent (see below, Resistance to Enzymes).

### Mode of Action

By thin section electron microscopy of phage enzyme-treated bacteria, it appears that the enzymes exert their lethal effects by digesting the peptidoglycan in localized areas, forming holes in the cell wall. Because the osmotic pressure inside a bacterium is approximately 3 atmospheres in relation to the normal external environment, this results in extrusion of the cytoplasmic membrane and, ultimately, hypotonic lysis. Since it has been found by Loessner [14] that these enzymes bind at an affinity of an immunoglobulin molecule, they are essentially one-use enzymes and require multiple molecules to accomplish the job of digesting the cell wall. One explanation as to why these molecules have evolved in this way is that after lysis the tight binding to the wall substrate will restrict the movement of enzyme molecules, preventing lysis of potential phage hosts nearby.

### Targeted Killing

An interesting feature of these enzymes is that they kill the species of bacteria from which they were produced. For instance, enzymes produced from streptococcal bacteriophage kill *streptococci*, and enzymes produced by pneumococcal bacteriophage kill *pneumococci *[6,7]. Specifically, the group C streptococcal lysin will kill group A *streptococci *efficiently and has a small effect on groups C and G *streptococci*, but has essentially no effect on normal oral *streptococci*. Similar results are seen with a pneumococcal-specific lysin. However in this case, the enzyme was also tested against strains of *pneumococci *that were resistant to penicillin, and the killing efficiency was the same. Unlike antibiotics, which are usually broad spectrum and kill many different bacteria found in the human body, some of which are beneficial, the phage enzymes only kill the disease bacteria with little to no effect on the normal human bacterial flora. Thus, phage lysins are molecules that enable targeted killing of pathogenic bacteria with little effect on the surrounding normal flora. When the group A streptococcal enzyme was tested for safety in two animal model systems, one mucosal and the other skin, in which enzyme was added to these surfaces daily for 7 days, and the tissues examined both visually and histologically, nothing unusual was observed. This was not surprising since the bonds cleaved by the phage enzymes are only found in bacteria and not human tissues. Thus, it is anticipated that these enzymes will be well tolerated in humans.

### In vivo Experiments

Two *in vivo *animal models of mucosal colonization were developed to test the capacity for the lysins to kill organisms on these surfaces. An oral colonization model was developed for group A *streptococci*, and a nasal model was developed for *pneumococci *[6,7]. In both cases, when the animals were colonized with their respective bacteria and treated with a small amount of lysin specific for the colonizing organism, the animals were found to be free of colonizing bacteria two to five hours after lysin treatment. In the group A streptococcal experiment, animals were also swabbed 24 and 48 hours after lysin treatment. During that time most animals remained negative for *streptococci*, but one animal had died and two others showed positive colonies. We interpret these results to mean that the positive animals became infected during the first four days of colonization, where some organisms became intracellular. Thus, while the lysin is able to clear organisms found on the surface, it was unable to kill organisms that had initiated an infection. We ruled out the possibility that the organisms that appeared at 24 and 48 hrs did so because they became resistant to the lysin by checking them for their sensitivity to the lysin.

### Controling Anthrax

Because phage enzymes are so efficient in killing pathogenic bacteria, they may be valuable tools in controlling biowarfare bacteria. To determine the feasibility of the approach, we identified a lytic enzyme from the gamma phage that is specific for *Bacillus anthracis *[15]. In cloning experiments using the gamma-phage DNA, we identified a ~700 bp ORF in the phage genome encoding a 26 kDa product very similar in size and features to a variety of *Bacillus *and *Listeria*, phage lysins. The gamma lysin, referred to as PlyG, was then purified to homogeneity by a two-step ion exchange chromatography procedure and tested for its lethal action on gamma phage sensitive *bacilli*. Three seconds after contact, as little as 10 ug of PlyG mediated a 5,000-fold decrease in viable counts of a ~10^7 ^*bacillus *culture [8]. This lethal activity was observed in growth media, phosphate buffer, and even human blood. When the enzyme was then tested against five mutant *B. anthracis *strains and ten different virulent *B. anthracis *strains isolated worldwide, it was found to be lethal for them all. Although PlyG has no effect on *B. anthracis *spores, we discovered that the addition of the germinant L-alanine after a short heat shock of 60C resulted in the rapid germination of the spore, at which time the enzyme was rapidly lethal.

When 10^7 ^*Bacillus cereus *strain RSVF (a close relative to *B. anthracis*) were administered intraperitoneally (IP) to fifteen mice, all died of rapidly fatal septicemia within four hours. When two sets of mice (n = 8 and n = 17) were also challenged IP with *bacilli *but given 150 μg and 50 μg of PlyG respectively 15 minutes later by the same route, 70%–80% of the animals survived. We anticipate that higher doses of enzyme or multiple doses will result in nearly 100% protection. In a separate experiment, two groups of mice were given 124 cfu of the *B. anthracis *Ames strain intravenously, and 15 minutes later one group received PlyG and the other buffer by the same route. When followed for 12 days, 90% of the enzyme treated mice survived, while only 10% of the control animals survived. Thus, we anticipate that this approach may be used in post-exposure cases of anthrax, in which individuals will be treated intravenously with PlyG to control the *bacilli *entering the blood after germination. This strategy could perhaps extend the window of antibiotic therapy and allow the innate immune system to clear any remaining *bacilli*.

### Resistance to Enzymes

Repeated exposure to low concentrations of lysin to bacteria grown on agar plates did not lead to the recovery of resistant strains, nor were we able to identify resistant bacteria after several cycles of exposure to low concentrations of enzyme in liquid [7]. This may be explained by the fact that the cell wall receptor for the pneumococcal lysin is choline [16], a molecule that is necessary for pneumococcal viability. For group A *streptococci*, we find that polyrhamnose, a cell wall component of the bacteria, is necessary for lysin binding (Nelson and Fischetti, unpublished), and polyrhamnose has also been shown to be important for streptococcal growth. While not yet proven, it is possible that during a phage's association with bacteria over the millennia, to avoid becoming trapped inside the host cell, the binding domain of their lytic enzymes has evolved to target a unique and essential molecule in the cell wall, making resistance to these enzymes a rare event.

### Immune Response to Enzymes

Because enzymes are proteins, one would anticipate an immune response to these molecules when delivered mucosally or systemically, resulting in the neutralization of the enzymatic activity. In order to test this, rabbits were hyperimmunized to the PlyG enzyme and the resulting antibodies tested in an ELISA assay. We found that in the two rabbits immunized, an antibody titer of >25,000 (reciprocal of the highest serum dilution yielding an OD of 1.0 in 30 min) was found for both antisera. When PlyG was diluted 1:1 with undiluted (or diluted) antisera or preimmune serum and tested for its lytic activity, no difference was observed in the lytic activity in the immune or preimmune sera. Thus, antibodies do not have the capacity to inactivate the PlyG, indicating that it may be used chronically or for extended periods. This does not appear to be a feature of the PlyG lysin alone, since similar results were observed with antibodies to the Cpl-1 enzyme directed to *pneumococci *[17].

## Conclusion

Phage enzymes have a broad application. Whenever there is a need to kill bacteria, and contact can be made with the organism, phage enzymes may be freely utilized. They may be used not only to control pathogenic bacteria on human mucous membranes, but may find utility in the food industry to control disease bacteria. Because of the serious problems of resistant bacteria in hospitals, day care centers, and nursing homes, particularly *staphylococci *and *pneumococci*, such enzymes may be of immediate benefit in these environments. Thus, we may add phage enzymes to our armamentarium against pathogenic bacteria. They are molecules that have been in development for millions of years by bacteriophage in their battle to survive within bacteria. They have yet to be exploited.

## Competing interests

The author(s) declare that they have no competing interests.
